# Identifying Barriers and Facilitators to the Improvement of Healthcare Delivery and Ethics in Two Cameroonian Neurosurgical Centers

**DOI:** 10.3389/fsurg.2021.703867

**Published:** 2022-02-15

**Authors:** Tutuwan J. Ankeambom, Mathieu Motah, Mfouapon Ewane, Nathan A. Shlobin, Celestin Bilong Mbangtang, Olaoluwa Ezekiel Dada, Kantenga Dieu Merci Kabulo, Francklin Tetinou, Geneviève Endalle, Ulrick Sidney Kanmounye, Luxwell Jokonya, Ignatius N. Esene

**Affiliations:** ^1^Faculty of Health Sciences, University of Buea, Buea, Cameroon; ^2^Department, Association of Future African Neurosurgeons, Yaounde, Cameroon; ^3^Neurosurgery Unit, Surgery Department, Douala General Hospital, Douala, Cameroon; ^4^Neurosurgery Unit, Surgery Department, Laquintinie Hospital, Douala, Cameroon; ^5^Faculty of Medicine and Biomedical Sciences, Yaounde, Cameroon; ^6^College of Medicine, University of Ibadan, Ibadan, Nigeria; ^7^Neurosurgery Unit, Surgery Department, Jason Sendwe General Provincial Hospital, Lubumbashi, Democratic Republic of Congo; ^8^College of Health Sciences, University of Zimbabwe, Harare, Zimbabwe; ^9^Neurosurgery Division, Faculty of Health Sciences, University of Bamenda, Bambili, Cameroon

**Keywords:** Cameroon, ethics, neurosurgery, barriers, health outcomes, facilitators

## Abstract

**Background:**

Low-and middle-income countries (LMICs) are disproportionately affected by neurosurgical burden of disease. This health inequity causes constraints in decision-making. Neurosurgical ethics helps us to assess the moral acceptability and effectiveness of clinical decisions. We aimed to assess ethical neurosurgical care and its effect on patient satisfaction in Cameroon.

**Methods:**

Two questionnaires hosted on Google Forms were administered among inpatients and staff at two Cameroonian neurosurgery centers. The questionnaires covered the factors influencing health outcomes and ethics. Data were collected from November 11, 2020, to March 11, 2021 and analyzed with SPSS v 26 to generate non-parametric tests with a threshold of significance at 0.05.

**Results:**

Seventy patients and twenty healthcare providers responded to the survey. Most patients faced financial hardship (57.1%; 95% CI = 45.7–68.6%), and felt that this affected the care they received (*P* = 0.02). Patients noticed changes in the care plan and care delivery attributable to the neurosurgical units' lack of resources. According to the patients and caregivers, these changes happened 31.0–50.0% of the time (42.9%, 95% CI = 5.7–21.4%). The majority of patients were pleased with their involvement in the decision-making process (58.6%; 95% CI = 47.1–70.0%) and felt their autonomy was respected (87.1%; 95% CI = 78.6–94.3%).

**Conclusion:**

Multiple challenges to neurosurgical ethical care were seen in our study. Multimodal interventions based on the four ethical principles discussed are necessary to improve ethical neurosurgical decision-making in this low resource setting.

## Introduction

The majority of low- and middle-income countries (LMICs) currently struggle to provide adequate neurosurgical services, with African countries disproportionately affected due to factors such as an insufficient number of neurosurgeons, inadequate healthcare infrastructure, and a paucity of equipment and funding (1). The neurosurgical workforce density in Africa is 1: 4,000,000 (2–4), while the average percentage of the population with access to neurosurgical services within a 2-h window is 25.3% in sub-Saharan Africa (5). These factors complicate the delivery of neurosurgical services to already underserved populations (6).

Challenges in decision-making arise due to a lack of standardized guidelines for neurosurgical techniques and management protocols, limited knowledge of surgical techniques, limited exposure to real time intraoperative decision making, and lack of guidelines regarding best practices for postoperative care (7, 8). Consideration of the ethical dimensions of decision-making is necessary to evaluate both the effectiveness and equitability of neurosurgical decisions, particularly as technology and care paradigms advance. Traditionally, ethical analyses are based on four key principles: respect for patient autonomy, beneficence, non-maleficence, and justice according to Beauchamp and Childress (9). Respect for patient autonomy refers to the right of the patient to make informed decisions about their medical care. The principle of beneficence is the obligation of physicians to act for the benefit of the patient, while non-maleficence involves avoiding the causation of harm. Justice refers to the balancing risks and benefits, ranging from the fair treatment of individuals to equitable allocation of healthcare resources (9).

To the best of our knowledge, no existing study has examined ethical decision-making within neurosurgery in an under-resourced setting. This study aims to assess ethical dimensions in the delivery of neurosurgical care in Cameroon and their relationship with patient satisfaction and outcomes. Our study will inform strategies for optimizing ethical decision-making in the delivery of neurosurgical care within the constraints of LMICs.

## Methods

### Study Setting

**Figure 1:**
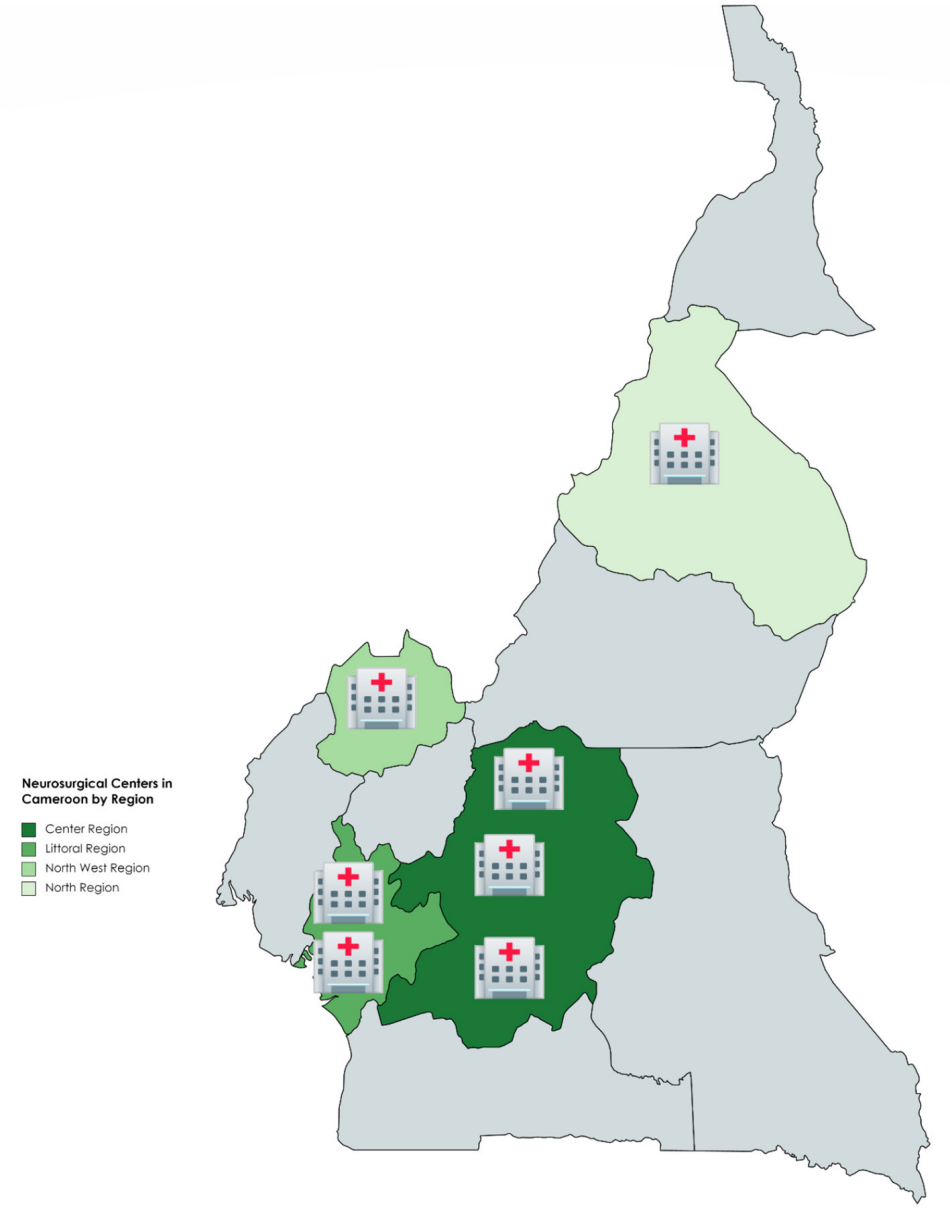
Neurosurgical Centers in Cameroon by Region.

**Figure 2:**
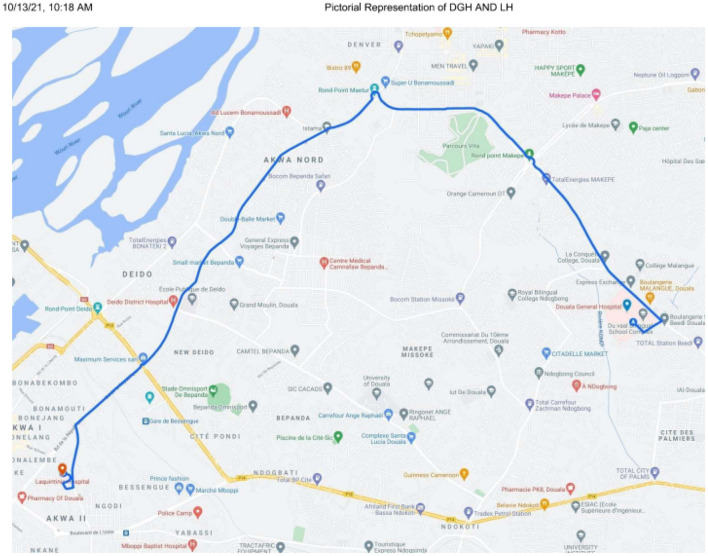
Pictorial Representation of DGH and LH.

This study was carried out in two Cameroonian neurosurgical centers—Laquintinie Hospital (LH) and Douala General Hospital (DGH). Figure 1 demonstrates the neurosurgical centers in Cameroon by region. In descending order, the Center Region has three: Yaounde General Hospital, Central Hospital Yaounde and Center Emergency Yaounde; Littoral Region:DGH and LH; North West Region: Bamenda General Hospital; North Region: Garoua Regional Hospital. The distance between Douala and Garoua, Bamenda and Yaounde in descending order is 1,338.4, 321.2, and 232.7 km, respectively. Figure 2 illustrates a pictorial representation of DGH and LH. The distance between DGH and LH is 10.2 km. Health facilities in Cameroon are organized according to categories, with category one as most well-equipped and category seven as least equipped. The level of equipment of each of these neurosurgical centers was based on the availability and maintenance of the equipment used to manage the neurosurgical pathologies common in this setting. Douala General Hospital is a category one hospital, while Laquintinie Hospital is a category 2 facility (10).

### Study Design

We conducted a cross-sectional survey among LH and DGH neurosurgery inpatients and personnel. This included patients who came to the surgical outpatient department for follow-up consultations after benefitting from surgical management of their neurosurgical disease and hospitalized patients in the postoperative period following a neurosurgical intervention. We did not include patients who were managed conservatively and medically for their neurosurgical diseases.

### Study Period and Duration

Data were collected from November 11, 2020, to March 11, 2021.

### Data Collection

Data were collected using Google Forms (Google LLC, California, USA) from November 11, 2020, to March 11, 2021.

Self-administered questionnaires designed in French and English were distributed among patients and healthcare workers who met the inclusion criteria. The patient questionnaire had six categories: sociodemographics (age, gender, level of education), resource allocation, variations in the standard of care, levels of satisfaction with the continuity of care and follow-up, cultural awareness, and disclosure of informed consent. The healthcare worker questionnaire had six categories: clinical roles, resource allocation, availability of resources, variations in the standard of care intraoperatively and postoperatively, continuity of care and follow-up, patient and procedure selection, cultural awareness, and disclosure and informed consent. The healthcare providers were also prompted to indicate other barriers and facilitators not mentioned in the questionnaire. Below is the patient and care provider's survey.

With respect to the ethical principles by Beauchamp and Childress, the survey was structured as follows: the third and fourth parts of the patient's survey evaluated maleficence and beneficence in neurosurgical care and the fifth part of the patient's survey measured autonomy. Justice was evaluated in parts of the second and third sections of the provider's survey.

Frequency, importance, and satisfaction were evaluated using Likert scales. Frequency was subdivided into: never (i.e., 0.0% of the time), occasionally (i.e., 1–30.0% of the time), sometimes (i.e., 31.0–50.0% of the time), usually (i.e., 51.0–80.0% of the time), and always (i.e., 81.0–100.0% of the time). Importance was subdivided into: very unimportant (0.0% importance), somewhat unimportant (1.0–30.0% importance), neutral (31.0–50.0% importance), somewhat important (51.0–80.0% importance), and very important (81.0–100.0% importance). Similarly, satisfaction was divided into very unsatisfied (0.0% satisfaction), unsatisfied (1–30% satisfaction), neutral (31–50% satisfaction), satisfied (51–80% satisfaction)n and very satisfied (81–100% satisfaction).

### Data Analysis

Data were analyzed with SPSS v 26 to generate non-parametric tests with a threshold of significance at 0.05. Categorical quantitative sociodemographic data and responses were computed as frequencies and percentages, while age was computed as a mean with a 95% confidence interval. Data regarding patient information, resource availability, service delivery, and patient satisfaction were compared between the hospitals (DGH vs. LH) and healthcare provider roles (physician vs. nursing and operating room staff). Responses to the open-ended questions were organized into themes by the first and senior authors. The interrater reliability (96.0%) was calculated using Cohen's kappa.

### Ethical Approval

The institutional review boards approved this study of DGH and LH (Ref 19AR/MINSANTE/HGD/DM/01/21 and 06394/AS/MINSANTE/DHL/CM). Patient consent for participation was obtained.

## Results

Seventy patients and twenty healthcare providers responded to the survey. This corresponds to response rates of 31.8 and 71.4%, respectively. The mean age of the patients was 40.6 (95% CI = 36.2–45.3) years. Most of them were male (74.3%; 95% CI = 64.3–84.3%), and few had attained tertiary education (17.1%; 95% CI = 8.6–27.1%). Half of the healthcare providers were nurses (50.0%; 95% CI = 30.0–70.0%) (Table 1).

**Table 1:** Sociodemographic characteristics of patients and healthcare providers that responded to the survey.

**Characteristics**	**Frequency (percentage; 95% confidence interval)**
**Patients (*****N*** **=** **70)**	
**Sex**	
Male	52 (74.3%; 64.3–84.3%)
Female	18 (25.7%; 15.7–35.7%)
**Education**	
Tertiary	12 (17.1%; 8.6–27.1%)
Secondary	34 (48.6%; 35.7–60.0%)
Primary	21 (30.0%; 20.0–41.4%)
No formal education	3 (4.3%; 0.0–10.0%)
**Healthcare providers (*****N*** **=** **20)**	
Nurse	10 (50.0%; 30.0–70.0%)
Neurosurgeon	5 (25.0%; 10.0–45.0%)
General practitioner	2 (10.0%; 0.0–25.0%)
Anesthetist	1 (10.0%; 0.0–15.0%)
Nurse assistant	1 (10.0%; 0.0–15.0%)
Operating room technician	1 (10.0%; 0.0–15.0%)

### Barriers to Care—Patient Perspective

**Figure 3:**
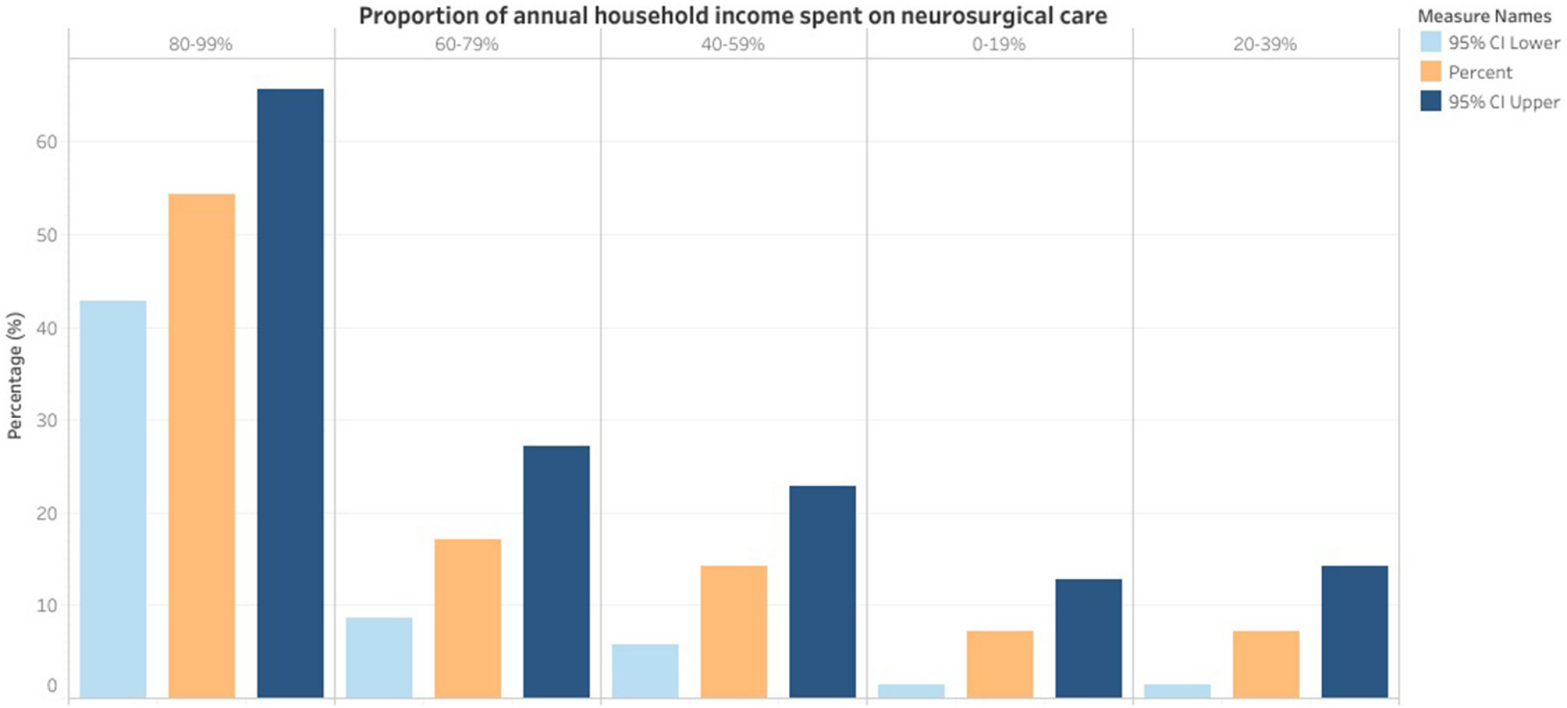
Proportion of the annual household income spent on neurosurgical care.

Most patients faced financial hardship (57.1%; 95% CI = 45.7–68.6%), and they felt this stress affected the care they received (*P* = 0.02). Similarly, more than half of the patients spent between 80 and 99% of their annual household income on neurosurgical care-related expenses (54.3%; 95% CI = 42.9–65.7%) (Figure 3). As a result, 82.9% (95% CI = 72.9–91.4%) had to borrow or crowdfund money for their neurosurgical care expenses. Patients at LH were less likely to face financial hardship (OR = 5.33; 95% CI = 1.16–18.04; *P* = 0.03) and to borrow or crowdfund for their health expenses (OR = 6.15; 95% CI = 1.48–21.00; *P* = 0.02).

The patients noticed changes in the care plan and care delivery attributable to the neurosurgical units' lack of resources. These changes referred to alterations in the initial care plan midway its execution due to a discovery that some resources were not there and an initial divergence from a preferred plan because of lack of resources. According to the patients and caregivers, these changes happened 31.0–50.0% of the time (42.9%, 95% CI = 5.7–21.4%). Only 20 patients (28.6%, 95% CI = 18.0–39.2%) got all the needed services at one of the study sites. The other patients had to go to another facility to get care. DGH patients were less likely to move from one hospital to another because of a lack of resources (25.0 vs. 32.4%; *P* = 0.01).

### Barriers to Care—Provider Perspective

Healthcare providers felt the greatest barriers to equitable access to care at the system level were lack of infrastructure (*n* = 13; 65.0%; 95% CI = 45.0–85.0%) and funding (*n* = 9; 45.0%; 95% CI = 25.0–65.0%) (Table 2).

**Table 2:** Barriers to equitable neurosurgical care.

**Characteristics**	**Frequency (percentage; 95% confidence interval)**
**Patient perspective**	
How often did lack of resources affect patient care?	28 (40.0%, 28.5–51.5%)
Never	4 (5.7%; 31.4–54.3%)
Usually	30 (42.9%; 5.7–21.4%)
Sometimes	4 (5.7%; 1.4–11.4%)
Occasionally	4 (5.7%; 1.4–11.4%)
**Always**	
**How often did you have to go to another health facility to get care?**	
Never	20 (28.6%, 18.0–39.2%)
Usually	10 (14.3%; 7.1–22.9%)
Sometimes	18 (25.7%; 15.7–35.7%)
Occasionally	12 (17.1%; 10.0–25.7%)
Always	10 (14.3%; 5.7–22.9%)
**Provider perspective**	
**Which of the following components of the neurosurgical system are**	
**barriers to equitable care?**	
Infrastructure	13 (65.0%; 45.0–85.0%)
Funding	9 (45.0%; 25.0–65.0%)
Workforce	6 (30.0; 9.9–50.0%)
Service delivery	4 (20.0; 2.5–37.5%)
Governance	4 (20.0; 2.5–37.5%)
Information management	1 (5.0%; 0.0–15.0%)

**Figure 4:**
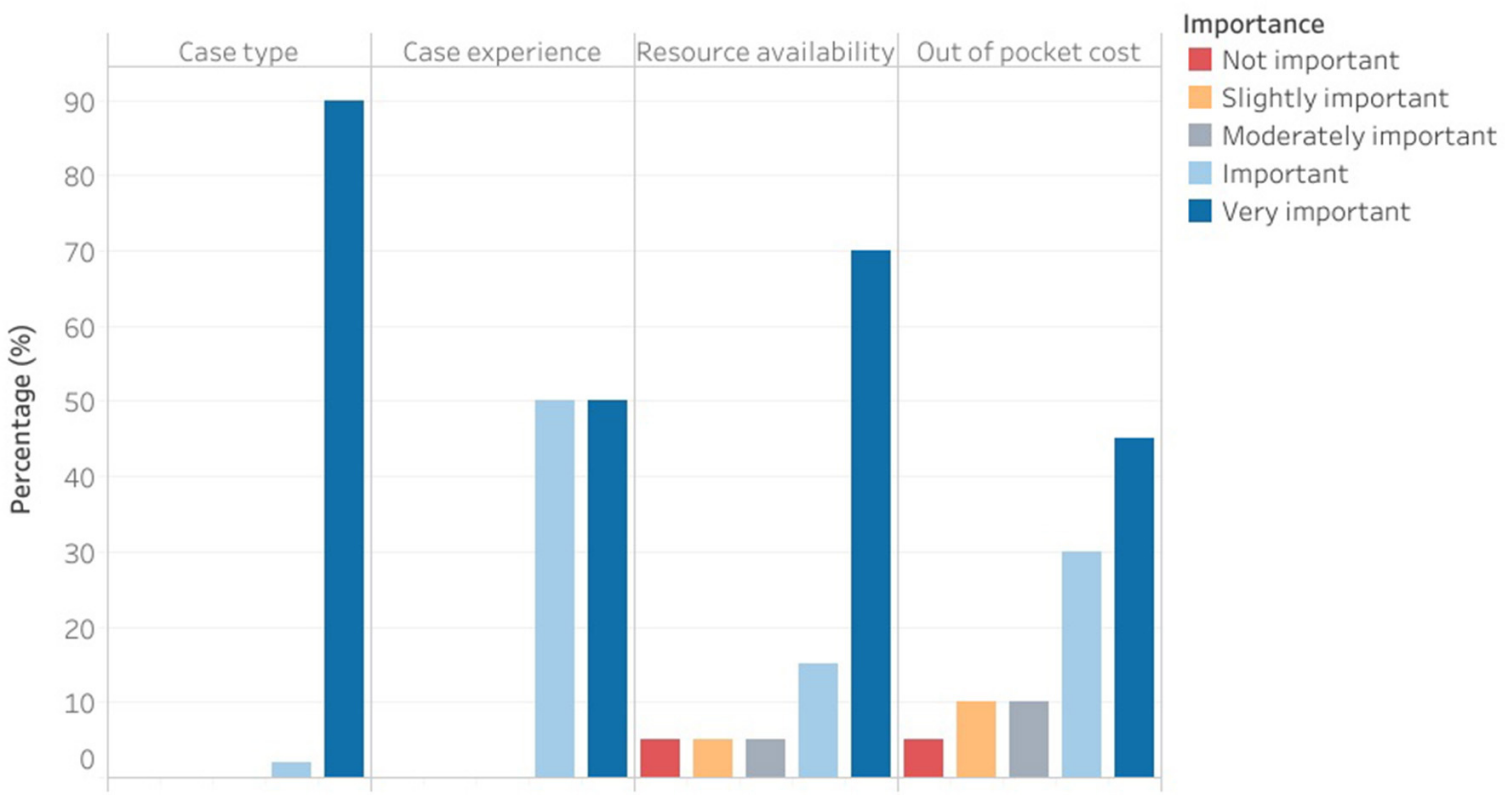
Factors influencing the quality of care and service delivery.

Suboptimal infrastructures influenced their decision-making by relegating their expertise and implementing the standard of care guidelines after resource availability considerations (80.0%; 95% CI = 62.5–97.5%). Other significant determinants of care delivery included: case type and experience (Figure 4).

### Patient Information

The majority of patients were pleased with their involvement in the decision-making process (58.6%; 95% CI = 47.1–70.0%) and felt their autonomy was respected (87.1%; 95% CI = 78.6–94.3%). Unfortunately, 51.4% (95% CI = 41.4–64.3%) did not receive enough information about the role and side effects of their prescribed medications. At discharge, only 70.0% (95% CI = 58.6–80.0%) of patients felt they had received comprehensive and digestible information on their management's next steps. Providers reported communicating information about the disease, therapeutic options, and therapy goals with patients and caregivers (85.0%; 95% CI = 70.0–100.0%). Despite sharing information with the patients and caregivers, healthcare providers reported communication challenges. These included fear of the unknown for patients and caregivers due to the uncertainty of the eventual outcome (55.0%; 95% CI = 35.0–75.0%), an inability for patients and caregivers to understand explanations (5.0%; 95% CI = 0.0–15.0%), and disagreements about the best course of action between patients and their families (5.0%; 95% CI = 0.0–15.0%).

### Patient Satisfaction

**Figure 5:**
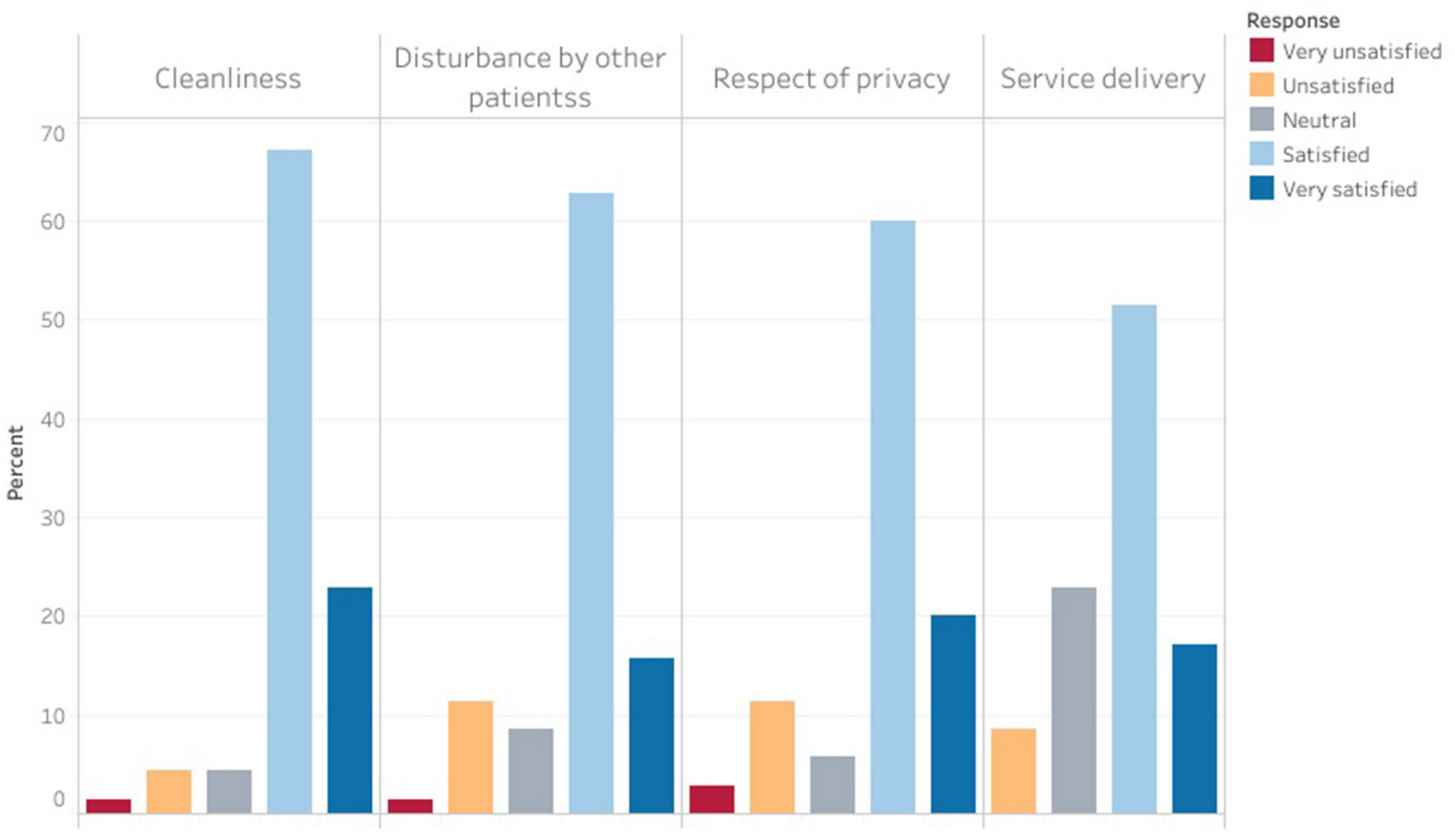
Neurosurgery patient satisfaction with service delivery and the care environment.

Patients and caregivers were generally satisfied with the service delivery and hospital environment (Figure 5).

Most providers reported regularly evaluating their patients (85.0%; 95% CI = 65.0–100.0%) especially in the postoperative period (90.0%; 95% CI = 75.0–100.0%). Also, they felt confident in their ability to recognize postoperative complications (95.0%; 95% CI = 85.0–100.0%).

With respect to the ethical principles by Beauchamp and Childress, we found that the principles of beneficence and maleficence were respected because most of the patients were satisfied with the service delivery and hospital environment. However, a good number of them felt their financial hardship affected the care they received. The principle of autonomy was respected as most of the patients were pleased with their involvement in the decision making process and most felt that their autonomy was respected.

Care providers had difficulties implementing the principle of Justice due to a lack of infrastructure and funding. In spite of this, they reported regularly communicating with their patients and following them up.

DGH patients were less likely to move from one hospital to another because most of the infrastructure and resources needed by the care providers were more available in DGH.

## Discussion

In this study, we identified the determinants of ethical service delivery in two Cameroonian neurosurgery centers. To the best of our knowledge, this is the first study examining ethical dimensions of neurosurgical care in LMICs. Some patients observed changes in the care plan and services delivered due to a lack of resources. As a result, a quarter of patients transferred from one health facility to another when imaging services and medications were unavailable at one of the two neurosurgical centers. The proportion of patients who were required to transfer was lower among patients at DGH than those at LH. This is because most of the infrastructure and resources needed by the care providers were more available in DGH.

Healthcare providers equally reported that lack of infrastructure and funding adversely impacted neurosurgical care.

### Information Provision and Informed Consent

Most patients did not understand the information they were given. This contrasted with the perception of healthcare providers, who reported communicating adequate information on the disease, therapeutic options, and treatment goals to patients and caregivers. Healthcare workers attributed unmet patient information needs to a lack of understanding on the part of patients. However, poor patient comprehension and low recall, accentuated further by low educational attainment, are likely responsible for this discrepancy (11, 12). Patient informational needs are frequently unmet, particularly regarding prognosis and follow-up after surgery and surgeon experience (13, 14). These findings highlight the need for neurosurgeons to remain aware of the discrepancy between their conceptualization of patient understanding and actual patient understanding, recognize common barriers to patient understanding, and attempt to minimize the effect of these barriers within the context of a patient encounter (11, 15).

Most patients reported their autonomy was respected, and were satisfied with their involvement in the decision-making process. Greater patient involvement in decision-making decreases patient anxiety and increases patient satisfaction (15). Current paradigms of patient involvement in neurosurgical decision-making focus on communication within consultations and the utilization of information conveyed by providers to decide regarding care (16). The informed consent process involves presenting the patient with information regarding their condition, possible treatments with associated risks and benefits, alternatives, and the risks and benefits of pursuing no treatment (17). Baseline patient health literacy and informational needs should guide discussions (17). Specialized interventions, such as specialized checklists for consent forms, question prompts, educational and interactive websites, and visual aids, are also necessary to facilitate patient understanding during the informed consent process (17). Augmented communication strategies, including providing quantifiable measures of success and risk, organizing the decision into a simple visual algorithm, utilizing methods to assess patient understanding such as teach-backs, and ensuring enough time for questions will optimize the informed consent process (17–19).

Together, these factors may improve patient understanding of their condition and treatment options, promoting patient-centered care and greater patient satisfaction. Importantly, informed consent is a continual process that requires continual information provision, assessment of patient understanding, and correction of misconceptions across the duration of care (17, 20, 21).

### Ethical Decision-Making in Low Resource Settings

Numerous strategies exist for improving ethical decision-making in low resource settings. Institutions in low resource settings can employ a series of simple yet far-reaching interventions. These include creating a set of ethical standards with clear guidelines regarding best practices, increasing awareness regarding common ethical issues, analyzing ethical examples and employing counterexamples to train trainees and neurosurgeons in how to conceptualize ethical decisions, basing the discussions of cases on objective information rather than value judgments, and using cases to continually refine the approach of neurosurgeons to ethical decision-making (22–24). A novel concept is appointing “ethical champions,” who are individuals in institutions who are responsible for creating ethical standards and maintaining ethical oversight of care practices, to actively guide ethical decision-making while maintaining consideration of limited resources (21). Ethical champions use “ethical frames” to increase team ethical awareness and reduce moral disengagement and “business frames” depending on the perceived effect of the ethical decision on factors such as resource allocation (21). In any case, ethical decision-making must hold principles of respect for patient autonomy, beneficence, non-maleficence, and justice as foundational.

### Limitations

This study had limitations. First, the study was conducted at the height of the COVID-19 pandemic, perhaps positively skewing patient responses given they received medical care during a challenging time. Second, we used a convenience sampling method which led to a small sample size, perhaps leading to excessive variability in survey responses. We also reported 95% confidence intervals to account for potential variability. Medical staff who did not respond were neuro-nurses and neuro anesthetists and patient caregivers may have been unwilling to participate given emotional stress, perhaps introducing non-response bias. In an effort to increase the response rate, we tried contacting the staff in person and on the phone at presumably convenient times and attempted to communicate clearly with caregivers and patients when they were visibly less stressed. Despite the aforementioned limitations, this study provides novel insight into ethical neurosurgical decision-making in Cameroon that may inform practices in other similar contexts.

## Conclusion

We identified multiple challenges to ethical neurosurgical care in two Cameroonian centers. Lack of resources affected service delivery, and patients had poor comprehension and recall of information conveyed to them by neurosurgeons. Specialized interventions are necessary to improve the informed consent process, while comprehensive measures to expand the application of ethical thinking to clinical encounters may improve ethical neurosurgical decision-making in low resource settings. These measures should be instituted across all levels of care delivery. Given the ethical challenges faced by providers in low-resource settings exhibit substantial overlap, our findings may guide best practices for ethical decision-making in LMICs.

## Data Availability Statement

The original contributions presented in the study are included in the article/supplementary material, further inquiries can be directed to the corresponding author/s.

## Author Contributions

All authors listed have made a substantial, direct, and intellectual contribution to the work and approved it for publication.

## Conflict of Interest

The authors declare that the research was conducted in the absence of any commercial or financial relationships that could be construed as a potential conflict of interest.

## Publisher's Note

All claims expressed in this article are solely those of the authors and do not necessarily represent those of their affiliated organizations, or those of the publisher, the editors and the reviewers. Any product that may be evaluated in this article, or claim that may be made by its manufacturer, is not guaranteed or endorsed by the publisher.
